# Delayed Effects of tDCS Combined with Cognitive Behavioral Therapy in Major Depression: A Randomized, Double-Blind Pilot Trial

**DOI:** 10.3390/brainsci15050444

**Published:** 2025-04-25

**Authors:** Sandra Carvalho, Catarina Gomes Coelho, Jorge Leite

**Affiliations:** 1 Psychological Neuroscience Laboratory, Centro de Investigação em Psicologia (CIPsi), Department of Basic Psychology, School of Psychology, University of Minho, 4710-057 Braga, Portugal; catarina-d@hotmail.com; 2 CINTESIS@RISE, CINTESIS.UPT, Portucalense University, 4200-072 Porto, Portugal; jorgel@upt.pt

**Keywords:** major depressive disorder, CBT, tDCS, neuromodulation, psychiatric symptoms, psychotherapy

## Abstract

Aims: This pilot study assessed the potential psychosocial and therapeutic impacts of augmenting transcranial direct current stimulation (tDCS) with cognitive behavioral therapy (CBT) in treatment-naïve patients diagnosed with major depressive disorder (MDD). Methods: In this double-blind randomized controlled trial, 10 subjects were randomized into two groups—CBT with active tDCS (active tDCS; *n* = 6; M = 33.3 years; 4 females) or CBT with sham tDCS (Sham; *n* = 4; M = 31.2 years; 2 females). Severity of depression was measured with the Montgomery–Åsberg Depression Rating Scale (MADRS) and the Beck Depression Inventory (BDI). Participants’ secondary outcomes included life satisfaction, sleep quality, and anxiety symptoms. They were assessed at baseline, following treatment (week 6), and at 2, 4, 8, and 12-week follow-ups. Results: By week 12, the active tDCS group’s BDI scores showed greater improvement relative to the sham group. There were also significant differences between groups over time in MADRS scores. Sleep quality also improved in the active tDCS group, with many participants achieving symptom-free status—defined as BDI scores of 9 or less and supported by consistently low MADRS scores—by the end of the follow-up period. Conclusions: These preliminary data indicate that the combination of tDCS with CBT may optimize the treatment of MDD through depressive symptom relief and improved sleep, while also prolonging the benefits of treatment.

## 1. Introduction

Major depressive disorder (MDD) is a common and disabling mood disorder that affects not only individual well-being but also social and occupational functioning. Relapse rates of 50% to 85% [1] highlight the heavy burden that MDD places on individuals and healthcare systems, underscoring the need for effective long-term management strategies. Beyond its emotional and psychological toll, MDD is a major contributor to morbidity and mortality, increasing the risk of suicide and a range of comorbid medical conditions [2]. Individuals with MDD often experience severe functional impairments, negatively affecting interpersonal relationships and overall quality of life. Depression is also linked to cardiovascular, metabolic, endocrine, and substance use disorders, all of which increase mortality risk and reduce life expectancy. In particular, depression is recognized as an independent risk factor for coronary heart disease (CHD). Studies indicate that individuals with depression are at a significantly higher risk of developing CHD, with anxiety and depression prevalence rates of 21% and 13%, respectively, in CHD patients [3]. The complex interplay between MDD and these medical comorbidities highlights the urgent need for integrated treatment approaches that address both mental and physical health to improve overall patient outcomes.

Recent evidence underscores the global burden of mental disorders among children and adolescents, emphasizing the early onset and persistence of these conditions. A comprehensive analysis based on Global Burden of Disease (GBD) 2019 data [4] estimates that approximately 293 million individuals aged 5 to 24 years—representing 11.63% of this population—experience at least one mental disorder. The prevalence rises with age, increasing from 6.80% in children (5–9 years) to 12.40% in early adolescence (10–14 years), 13.96% in mid-adolescence (15–19 years), and 13.63% in young adulthood (20–24 years). These results underscore the urgent need for early interventions to prevent lasting psychological and functional impairments. Given the high prevalence of depression within this demographic, developing innovative, scalable, and accessible therapeutic approaches remains a pressing global mental health priority.

Despite extensive research and treatment options, response rates for MDD remain unsatisfactory. Although pharmacotherapy and cognitive behavioral therapy (CBT) are considered first-line treatments, recent evidence suggests their effectiveness may be overestimated due to factors like publication bias and researcher allegiance. For instance, studies have found that the efficacy of psychological interventions for depression has been overestimated in the published literature, just as it has been for pharmacotherapy [5]. Additionally, researcher allegiance has been identified as a potential source of bias in randomized clinical trials of psychological interventions [6]. Moreover, head-to-head comparisons of psychotherapies versus pharmacotherapies reveal negligible differences, while combined treatment offers only a modest benefit over monotherapy [7]. These findings highlight the urgent need for novel or adjunctive therapeutic strategies to enhance treatment outcomes.

One promising alternative intervention is transcranial direct current stimulation (tDCS). tDCS is a non-invasive brain stimulation technique that modulates cortical excitability and has shown promise in alleviating depressive symptoms, currently holidng a level of evidence A [8]. Growing evidence from randomized controlled trials and meta-analyses suggests that tDCS may serve as an effective adjunct to conventional treatments for MDD. Meta-analyses have further corroborated the efficacy of tDCS in treating depression. A review of several randomized trials found that tDCS significantly reduces depressive symptoms, especially when delivered at 2 mA for 30 min sessions [9]. Additionally, tDCS has demonstrated the potential to reduce suicidal ideation and provide sustained antidepressant effects [10]. Studies have shown that targeting the left DLPFC can significantly improve depressive symptoms for a month or longer, indicating the durability of tDCS benefits. These findings position tDCS as a promising alternative or adjunctive intervention for MDD, particularly for patients who have not responded adequately to traditional therapies.

One of the key neurobiological rationales for using tDCS in MDD is based on electrophysiological evidence of frontal alpha asymmetry observed in resting electroencephalogram (EEG) recordings. Individuals with MDD often exhibit reduced activity in the left dorsolateral prefrontal cortex (DLPFC) relative to the right, a pattern associated with emotional dysregulation and depressive symptomatology [11]. This asymmetry may increase vulnerability to depression, with left DLPFC activity linked to approach motivation and right DLPFC activity to withdrawal and avoidance [12]. Recent research has examined the correlation between frontal alpha asymmetry (FAA) and MDD. A 2023 meta-analysis of 23 studies (1928 MDD patients, 2604 controls) assessed frontal alpha asymmetry (FAA) as a possible biomarker for MDD. The research showed that individuals with MDD displayed increased right frontal EEG alpha asymmetry relative to non-depressed individuals, indicating that frontal alpha asymmetry may function as a biomarker for MDD [13]. The findings corroborate the idea that diminished activity in the left dorsolateral prefrontal cortex (DLPFC) compared to the right is linked to emotional dysregulation and depressive symptoms. This imbalance may predispose individuals to depression, as heightened left DLPFC activation coincides with approach desire, whereas increased right DLPFC activity is linked to withdrawal and avoidance tendencies.

By delivering a low-intensity direct current (typically 1–2.5 mA) through scalp electrodes for 10–30 min, tDCS can depolarize or hyperpolarize neuronal membranes, effectively modulating cortical excitability [14]. The most common electrode montage involves placing the anode (excitatory) over the left DLPFC and the cathode (inhibitory) over the right DLPFC or positioning the anode over the left DLPFC and the cathode over the right supraorbital region, therefore restoring the inter-hemispheric imbalance [15]. Although tDCS has proven effective in reducing MDD symptoms, its potential as an adjunct to psychotherapy remains underexplored. Because both CBT and tDCS influence prefrontal regulation of emotion and cognition, combining them has a strong theoretical foundation for enhancing treatment outcomes. A study protocol by Carvalho et al. (2020) [15] proposed investigating the clinical and mechanistic effects of combining CBT and tDCS in MDD treatment. Building on this foundation, the present study aims to investigate the repetitive effects of tDCS combined with individualized CBT in alleviating depressive symptoms in individuals with mild to moderate MDD who are CBT-naïve. We hypothesize that combining tDCS with CBT will have a synergistic effect, improving CBT’s effectiveness and resulting in better clinical outcomes than CBT alone. Additionally, we aim to examine the long-term effects of these interventions, assessing their sustained impact over a three-month follow-up period.

Taken together, these findings highlight the necessity for comprehensive, evidence-informed therapy approaches that target both the neurobiological and psychosocial foundations of depression. This study aimed to assess the possible synergistic benefits of combining tDCS and CBT, both of which target prefrontal cortex function, in patients with mild to severe, treatment-naïve MDD. We propose that including active tDCS into personalized CBT will result in more significant reductions in depressive symptoms, as well as enhancements in sleep, anxiety, and life satisfaction, in contrast to CBT combined with sham stimulation. Moreover, we anticipate that the therapeutic effects would endure and even intensify during a 12-week follow-up period. This pilot trial seeks to furnish initial information about the efficacy and longevity of this combination intervention and to guide the formulation of more effective and scalable treatment models for MDD.

## 2. Materials and Methods

### 2.1. Ethical Approval and Study Registration

This study was conducted in accordance with the latest revision of the Declaration of Helsinki and received ethical approval from the Subcommittee on Ethics for Life and Health Sciences (SECVS) at the University of Minho (Approval No. SECVS 174/2017). All participants provided written informed consent prior to enrollment. The study protocol was registered with the United States National Library of Medicine Clinical Trials Registry (ClinicalTrials.gov ID: NCT03548545) on 7 June 2018 (Protocol Version 1). Further methodological details are available in the published protocol by Carvalho et al. (2020) [15]: clinicaltrials.gov/ct2/show/NCT03548545 (accessed on 25 April 2023).

### 2.2. Participant Recruitment and Eligibility Criteria

Participants were recruited through multiple sources, including social media advertisements, flyers distributed throughout the city of Braga, and institutional mailing lists from the University of Minho. Eligibility was restricted to individuals experiencing an acute major depressive episode who met the Diagnostic and Statistical Manual of Mental Disorders (DSM-5) criteria for MDD. To ensure a well-characterized sample, the following inclusion criteria were applied: participants had to be between 18 and 75 years of age, present a diagnosis of unipolar, non-psychotic MDD, and score ≥7 on the Montgomery-Åsberg Depression Rating Scale (MADRS) and ≥10 on the Beck Depression Inventory (BDI). Additionally, all participants were required to demonstrate low suicide risk, which was assessed using the Structured Clinical Interview for DSM (SCID). Although the SCID-5 version was not yet available to our team at the time of data collection, the structured interview was conducted by a trained clinician, and all diagnoses and risk assessments were confirmed according to DSM-5 criteria. As a supplementary measure, suicide risk was also evaluated using Item 9 of the BDI, which specifically addresses suicidal thoughts. Exclusion criteria encompassed any contraindications to transcranial direct current stimulation (tDCS), such as the presence of metal implants in the head or implanted cranial medical devices. Participants were also excluded if they presented with any significant or unstable neurological or psychiatric conditions other than MDD, including but not limited to epilepsy, Parkinson’s disease, dementia, eating disorders, or obsessive-compulsive spectrum disorders. Additional exclusion criteria included a lifetime history of substance use disorders, a diagnosis of a personality disorder, or any severe medical condition likely to impair functional status during the study period (e.g., active cancer or serious cardiac, renal, or hepatic conditions). Although the original study protocol excluded individuals receiving psychopharmacological treatment, an amendment was implemented to include participants undergoing stable pharmacotherapy. This adjustment was made to enhance the external validity and clinical relevance of the findings, while maintaining methodological integrity.

### 2.3. Study Design

**Figure 1:**
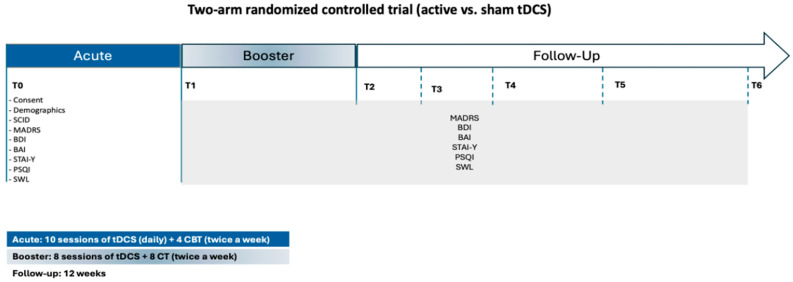
Study design and assessment timeline of the two-arm randomized controlled trial (RCT), comparing active vs. sham transcranial direct current stimulation (tDCS), combined with cognitive behavioral therapy (CBT).

This randomized, double-blind, controlled pilot trial was designed to assess the therapeutic efficacy of combining tDCS with CBT in adults experiencing an acute episode of MDD. The intervention spanned a total duration of six weeks. Participants were randomly allocated to one of two conditions: active tDCS paired with CBT or sham tDCS paired with CBT. Each participant completed 18 sessions of tDCS (active or sham) and 12 sessions of CBT, implemented in accordance with a standardized treatment protocol (Figure 1).

The intervention was structured into two consecutive phases. The acute treatment phase, corresponding to the first two weeks, consisted of 10 consecutive weekday sessions of tDCS (Monday to Friday). During this period, no CBT sessions were administered. The reinforcement phase, covering weeks three to six, incorporated two weekly tDCS sessions (on Mondays and Fridays), each lasting 30 min. CBT was introduced during this phase, with participants attending a total of four individual therapy sessions delivered by a licensed clinical psychologist. Clinical assessments were conducted at seven key time points: baseline (prior to randomization), end of the acute phase (week 2), end of the reinforcement phase (week 6), and at four post-intervention follow-up intervals (2, 4, 8, and 12 weeks). This schedule allowed for the evaluation of both immediate and sustained treatment effects. All study procedures—including clinical evaluations, neuromodulation sessions, and psychotherapy—were carried out at the Clinical Service of the School of Psychology, University of Minho. Participants were referred by Primary Care Physicians, ensuring a clinically representative cohort of individuals actively seeking treatment for depressive symptoms.

### 2.4. Randomization and Blinding Procedures

Randomization procedures followed the protocol outlined by Carvalho et al. (2020) [15]. After confirming eligibility and obtaining written informed consent, participants were randomly assigned to one of two intervention arms—active tDCS combined with CBT or sham tDCS combined with CBT—using a computer-generated, web-based randomization system. A 1:1 allocation ratio was applied to ensure balanced group distribution. To preserve allocation concealment and ensure methodological rigor, the randomization sequence was stored in sealed, opaque envelopes and accessed only at the time of assignment. Blinding was maintained across multiple levels of the study; participants, CBT therapists, clinical assessors, and data analysts were all blinded to group allocation throughout the trial. Due to the technical requirements of stimulation delivery, the research staff responsible for administering tDCS were not blinded, as they were required to implement specific stimulation parameters. No serious adverse events were observed during the course of the study. In the event of an adverse event requiring unblinding, the Principal Investigator was authorized to disclose the participant’s group allocation and report the incident to the Ethics Committee within 24 h, in accordance with institutional and ethical safety protocols.

### 2.5. Screening and Baseline Assessment

Individuals who expressed interest in participating were initially screened through a structured intake process to evaluate eligibility for study inclusion. During this initial contact, a standardized questionnaire was administered to assess compliance with the inclusion and exclusion criteria, as well as suitability for transcranial direct current stimulation (tDCS) and electroencephalogram (EEG) procedures. Potential participants were fully informed of the study’s aims, procedures, and potential risks, and had the opportunity to ask questions prior to providing written informed consent. Eligible participants were then scheduled for the baseline assessment (T0). During this session, participants completed standardized demographic and medical history questionnaires, followed by administration of the primary clinical outcome measures: the Montgomery-Åsberg Depression Rating Scale (MADRS) and the Beck Depression Inventory (BDI). Individuals who met the eligibility criteria based on these assessments proceeded to complete the secondary outcome measures, including the Beck Anxiety Inventory (BAI), State-Trait Anxiety Inventory (STAI-Y), Pittsburgh Sleep Quality Index (PSQI), and the Satisfaction with Life Scale (SWLS). To confirm the presence of MDD according to DSM-5 criteria, participants underwent a Structured Clinical Interview for DSM-5 (SCID-5), conducted by a trained clinician. The entire baseline assessment protocol required approximately two hours to complete.

### 2.6. Intervention Phases and Follow-Up Assessments

Following the baseline assessment (T0), participants entered the acute treatment phase, which spanned two weeks. During this phase, individuals received 10 consecutive weekday sessions of transcranial direct current stimulation (tDCS), administered Monday through Friday. Each session consisted of either active or sham stimulation at 2 mA for 30 min. In parallel, participants began cognitive behavioral therapy (CBT), attending two sessions per week (Monday and Friday), totaling four sessions during this initial phase. At the end of the acute treatment phase (T1; week 2), participants underwent a comprehensive reassessment that included the same battery of primary and secondary clinical outcome measures administered at baseline. Participants then transitioned into the reinforcement phase, which covered weeks 3 to 6. During this period, they continued to receive two tDCS sessions per week (30 min each, on Mondays and Fridays), alongside CBT sessions at the same frequency. Upon completing this phase (T2; week 6), participants underwent a post-treatment evaluation, using the same clinical outcome measures. In addition to clinical assessments, three-minute resting-state EEG recordings were obtained at T0, T1, and T2. As EEG analyses fall outside the scope of the present manuscript, those data will be reported in a separate publication. To evaluate the durability of treatment effects, participants were further assessed at four follow-up time points: at 2 weeks (T3), 4 weeks (T4), 8 weeks (T5), and 12 weeks (T6) after the conclusion of the intervention. This follow-up period enabled a longitudinal evaluation of symptom trajectories and maintenance of therapeutic outcomes.

### 2.7. Cognitive Behavioral Therapy (CBT) Protocol

The cognitive behavioral therapy (CBT) component of the intervention was structured in accordance with Aaron T. Beck’s cognitive theory of depression and delivered following evidence-based clinical guidelines. Each session lasted approximately 60 min and was conducted individually to allow for personalized therapeutic engagement. The intervention was tailored to the severity and specific symptom profile of each participant, with an emphasis on identifying, evaluating, and restructuring maladaptive cognitive patterns, dysfunctional beliefs, and associated behavioral responses contributing to depressive symptomatology. Therapeutic strategies included cognitive restructuring, behavioral activation, thought monitoring, and emotion regulation techniques. Sessions also incorporated psychoeducation regarding the cognitive model of depression and relapse prevention strategies in later phases of treatment. The therapeutic process was designed to enhance participants’ coping skills, promote insight into negative automatic thoughts, and foster the development of adaptive problem-solving approaches. All CBT sessions were delivered by a licensed clinical psychologist registered with the *Ordem dos Psicólogos Portugueses* (OPP—the official regulatory body for the psychology profession in Portugal), with formal training in cognitive behavioral therapy. To ensure consistency, fidelity to the treatment model, and adherence to protocol guidelines, weekly supervision meetings were held between the treating psychologist and a senior clinical supervisor (SC), who possessed extensive clinical experience in CBT for mood disorders. Supervision focused on case conceptualization, treatment planning, session content, and addressing therapeutic challenges, thereby ensuring the quality and integrity of the intervention across participants.

### 2.8. Transcranial Direct Current Stimulation (tDCS) Administration

Transcranial direct current stimulation (tDCS) was administered using the Eldith DC Stimulator Plus (NeuroConn GmbH, Ilmenau, Germany), a CE-certified device widely employed in clinical and experimental neuromodulation research. Stimulation was delivered through two saline-soaked sponge electrodes (25 cm^2^ each), secured using elastic headbands to ensure consistent placement and contact. Electrode montage followed the international 10–20 EEG system: the anodal electrode was positioned over the left dorsolateral prefrontal cortex (DLPFC), at site F3, and the cathodal electrode over the right DLPFC, at site F4. This bilateral frontal configuration is supported by neurophysiological evidence linking prefrontal asymmetry to affective regulation and has been frequently used in studies targeting depressive symptoms. In the active tDCS condition, participants received a constant current of 2 mA (resulting in a current density of 0.80 A/m^2^) for a total duration of 30 min. Stimulation included a 15 s ramp-up and ramp-down to minimize discomfort and prevent sudden perceptual sensations associated with abrupt current onset or offset. In the sham condition, the electrode montage and ramp-up/down parameters were identical to those of the active condition; however, the device automatically discontinued stimulation after 15 s of 2 mA current. This brief stimulation period is considered sufficient to mimic the initial tingling sensation experienced in active stimulation, thereby preserving the integrity of the blinding protocol. Each tDCS session lasted approximately 40 min, accounting for preparation, electrode placement, and stimulation time. All sessions were conducted in a quiet, controlled environment, with participants seated comfortably in a semi-reclined position to minimize movement and ensure optimal electrode contact. Importantly, tDCS was administered by a trained research assistant who was not involved in any aspect of clinical assessment or cognitive behavioral therapy delivery. This procedural separation was implemented to preserve the double-blind design, ensuring that therapists, assessors, and participants remained blinded to the stimulation condition throughout the study.

### 2.9. Statistical Analysis

Given the small sample size and the non-normal distribution of the data in this pilot study, only non-parametric statistical analyses were performed. To explore potential group differences in sociodemographic characteristics between the active and sham tDCS groups, Chi-squared tests were used for categorical variables, while the Mann–Whitney U test was applied to continuous variable. Group differences in psychiatric symptoms across the seven assessment time points were analyzed using the Mann–Whitney U test, with effect sizes calculated using the rank-biserial correlation.

Additionally, a secondary analysis was conducted to explore symptom improvement over time for each group relative to baseline. We calculated group-normalized change scores; each participant’s symptom scores at time points T1 to T6 were divided by the average symptom score of their respective group at T0. This approach allowed us to examine within-group improvement patterns and between-group differences while accounting for initial baseline variability. The Mann–Whitney U test was used for group comparisons, with effect sizes calculated using the rank-biserial correlation (r).

To supplement continuous outcome analyses, we also computed clinically meaningful categorical outcomes. For the MADRS, a reduction of ≥12 points from baseline was defined as a clinically meaningful treatment response, in line with established guidelines [13]. For the BDI, a score of ≤9 was used as a cutoff to define remission, based on prior validation studies [16]. Participants were categorized accordingly (Yes/No), and differences between groups at each time point were tested using Chi-squared tests. This approach enabled an evaluation of both symptom trajectories and clinically relevant treatment milestones.

Bivariate correlations between symptoms were analyzed at each assessment time using Spearman’s correlation coefficient. Descriptive statistics, group differences, and correlation analyses were carried out using IBM SPSS Statistics 23. Additionally, all result graphs and effect size calculations were performed using R software, version V 4.4.2.

## 3. Results

### 3.1. Sociodemographic Data

Therefore, from the 13 (*n* = 13) participants who were initially screened, 1 participant was excluded due to regular substance use, 1 participant was excluded due to the additional diagnosis of Tourette syndrome, and 1 participant was excluded because they started taking psychopharmacological treatment with sertraline.

Ten adults diagnosed with MDD participated in this clinical trial (age: *Mdn* = 31, *IQR* = 20.5; 60% female). Participants were allocated into either the active group (*n* = 6) or the sham group (*n* = 4). Overall, 60% of participants had completed high school education, 80% were single, and 50% were employed at the time of the first assessment. None of the participants met diagnostic criteria for additional psychiatric disorders aside from major depressive disorder, and no psychiatric comorbidities were present at baseline. All participants were also screened for suicide risk using the Structured Clinical Interview for DSM (SCID), which confirmed low risk in all cases.

Table 1 shows the sociodemographic characteristics of participants, comparing both the active and sham groups. All variables presented non-significant differences between the two groups.

### 3.2. Groups Differences in Psychiatric Symptoms over Time

**Figure 2:**
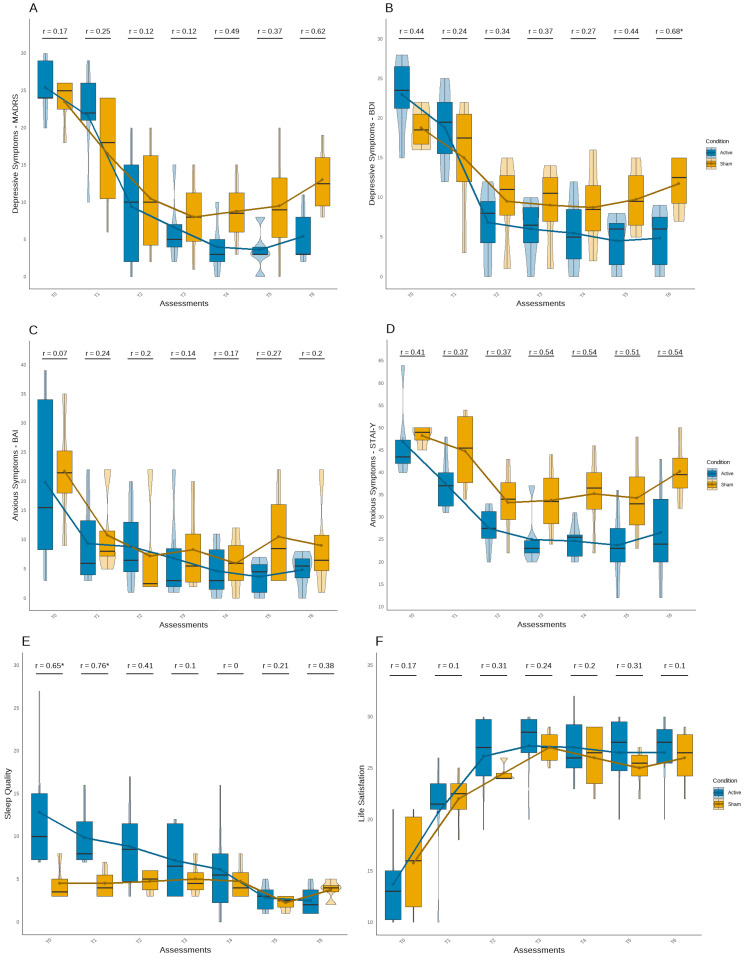
Symptom trajectories across treatment and follow-up phases for both groups. Symptom trajectories across assessments for the active (blue) and sham (yellow) groups. Panels show: (**A**) MADRS, (**B**) BDI, (**C**) BAI, and (**D**) STAI-Y scores from T0 to T6, (**E**) Sleep Quality, and (**F**) Life Satisfaction scores from T0 to T6. Black lines represent group trends. Pearson’s r values indicate symptom change over time; *p* < 0.05 is marked with *.

Based on the analysis of differences between the active and sham tDCS groups over time (Figure 2), symptom improvement was observed in both groups. As expected, the active tDCS group showed greater long-term benefits for most psychological symptoms.

Although no significant differences were observed in depression symptoms until the last follow-up assessment (U = 2.00, Z = −2.15, *p* < 0.05) (Figure 2A,B), the active tDCS group started with higher mean scores on both instruments. From T2 assessment, this group presented fewer symptoms compared to the sham group, showing scores below the cut-offs for minor depressive symptoms (MADRS < 6; BDI < 9). The effect sizes also suggested a stronger and more consistent group difference at the end of the follow-up period in both questionnaires.

Despite no significant differences being observed in anxiety symptoms (Figure 2C,D), an interesting but non-significant pattern was observed in the STAI-Y1 (Figure 2D), in which the active tDCS group maintained a decrease in symptoms across all post-baseline assessment points until the end of the experiment, with medium to large effect size values. Although sleep quality scores were better in the sham tDCS group at baseline and T1, with a significant difference (U = 2.50, Z = −2.04, *p* < 0.05; U = 1.00, Z = −2.39, *p* < 0.05), the active tDCS group showed a continuous improvement in sleep quality throughout the experiment, reporting fewer sleep difficulties by the final follow-up assessment, although the difference was not statistically significant (Figure 2E). Additionally, both groups demonstrated an improvement in life satisfaction, with no significant group differences or robust effect sizes.

To complement these analyses, we conducted additional categorical assessments based on established clinical cutoffs. Participants were categorized as responders if they showed a ≥12-point reduction in MADRS scores from baseline, and as remitters if they had BDI scores ≤ 9, consistent with previous studies [13,16].

**Figure 3:**
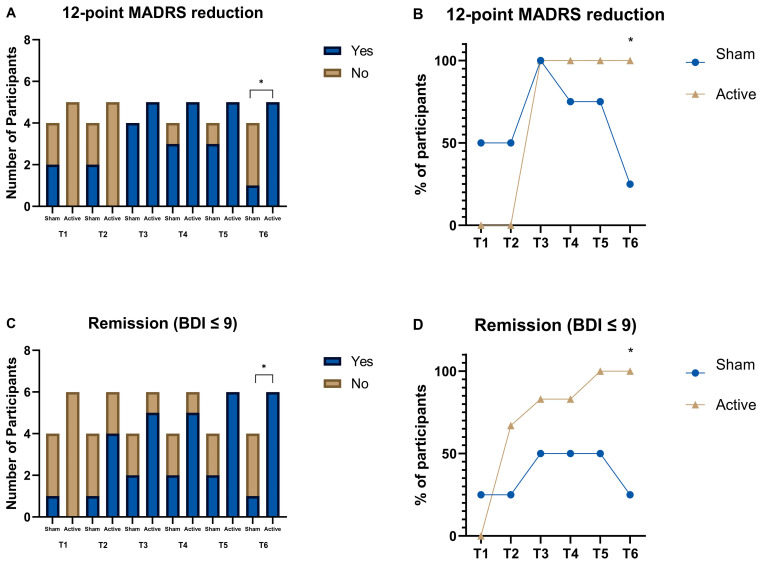
Response and remission rates over time by treatment group. (**A**) Number of participants achieving a ≥12-point reduction in MADRS scores and (**B**) corresponding percentage across timepoints (T1–T6), for active (gold) and sham (blue) conditions. (**C**) Number of participants reaching remission criteria (BDI ≤ 9) and (**D**) corresponding percentages across timepoints. Asterisks (*) indicate significant group differences at T6 (*p* < 0.05).

Despite the small sample size, we performed two additional analyses (Figure 3). The first analysis was based on a 12-point reduction in the MADRS from baseline, a threshold associated in the literature with substantial symptom change [13]. Data were categorized as Yes or No, and Chi-squared tests were performed for each time point. On the last follow-up (T6), there was a substantial difference in scores between those who received active versus sham tDCS (χ^2^ (1, *n* = 9) = 5.625, *p* = 0.018) (Figure 3A,B). Similarly, when analyzing BDI scores using a cutoff point of 9 as an indicator of remission [16], participants in the active tDCS group showed higher remission rates than those in the sham group at the final follow-up (χ^2^ (1, *n* = 10) = 6.429, *p* = 0.011) (Figure 3C,D). Figure 3 illustrates the proportions of participants who met response and remission criteria at each time point. At the final follow-up (T6), the active tDCS group had significantly higher rates of both response (≥12-point reduction in MADRS; χ^2^ (1, *n* = 9) = 5.625, *p* = 0.018) and remission (BDI ≤ 9; χ^2^ (1, *n* = 10) = 6.429, *p* = 0.011) compared to the sham group. A similar trend toward higher remission was already apparent at the 8-week follow-up (T5) (χ^2^ (1, *n* = 10) = 3.750, *p* = 0.053). These results support the added clinical benefit of combining tDCS with CBT, suggesting that the therapeutic effects may become more pronounced over time.

### 3.3. Psychiatric Changes over Time in Relation to Baseline

**Figure 4:**
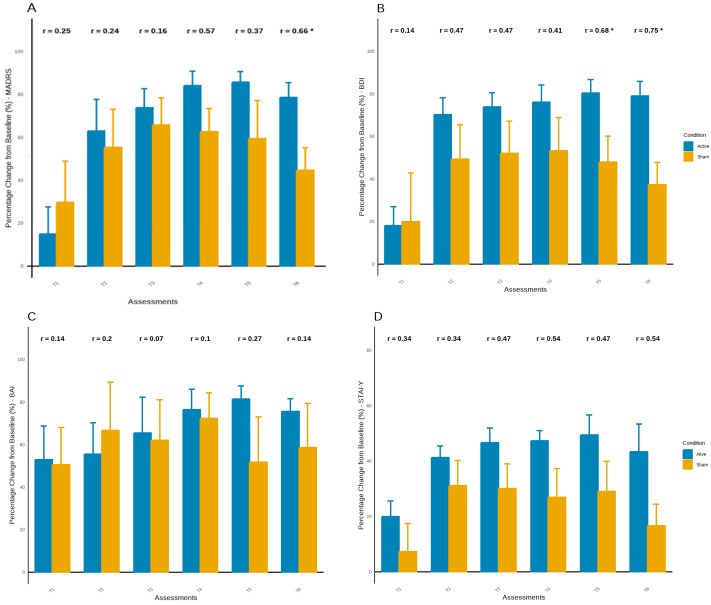

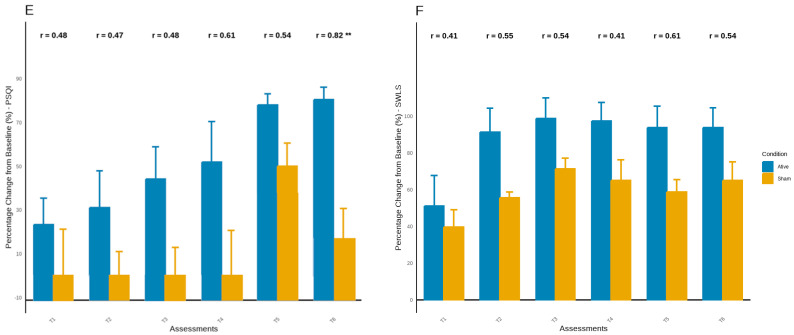
(**A**–**F**) Percentage change from baseline in clinical outcomes across assessment points. Error bars represent standard error. Pearson’s r values indicate correlations between time and symptom reduction or improvement within each group; *p* < 0.05 is marked with an asterisk (*), *p* < 0.01 is marked with an asterisk (**).

The percentage change in symptoms across each assessment relative to T0 showed consistent improvement in most symptoms in both groups. However, this improvement appears to be more consistent in the active tDCS group, mainly in the latest assessments (Figure 4). Before the 8 week follow-up (T5), the active tDCS group demonstrated an improvement of over 85%, while the sham group improved by approximately 50% to 60%, with significant between-group differences observed in BDI scores (U = 2.00, Z = −2.15, *p* < 0.05) (Figure 4A,B). Similarly, at the 12 week follow-up (T6), the active tDCS group showed an improvement of about 80% in depressive symptoms, while the sham group improved by about 40%, with significant between-group differences found in the MADRS and BDI scores (U = 2.00, Z = −1.97, *p* < 0.05; U = 1.00, Z = −2.37, *p* < 0.05) (Figure 4A,B). Thus, in addition to the higher improvement in depressive symptoms observed in the active group, this group maintained better results until the last assessment (T6), whereas the sham group exhibited a symptom rebound of more than 20%.

Although no significant findings were observed in anxiety symptoms, results suggest greater improvement in the active tDCS group, with increasing effect sizes between groups during follow-up assessments, mainly in STAI (Figure 4C,D). Additionally, the active tDCS group showed continuous improvement in sleep quality until the last assessment, significant difference at the final time point, and an improvement of over 80% from baseline (*p* < 0.05). In contrast, the sham tDCS group ended the experiment with an improvement of around 15% (Figure 4E). No significant findings were observed in life satisfaction, however the active tDCS group maintained higher levels compared to the sham group until the end of experiment, with higher effect sizes (Figure 4F). No significant findings were observed in life satisfaction. However, the active tDCS group maintained higher levels compared to the sham group until the end of the experiment, with larger effect sizes (Figure 4F).

### 3.4. Correlations in Psychiatric Symptoms over Time

The subsequent correlations among symptom dimensions are characterized as exploratory and descriptive owing to the non-independence of repeated assessments. These findings offer first insights but must be interpreted cautiously, particularly considering the limited sample size and the repeated evaluations over time. The correlations between symptoms emerged from the last assessment after the intervention (Table 2). In addition to the positive relationship between changes in depressive and anxiety symptoms during these assessments (*p* < 0.001 to *p* < 0.05), a negative relationship was found at T2 between changes in life satisfaction perception and anxiety levels (*p* < 0.05). Two weeks after the intervention, a positive relationship was observed between changes in depressive symptoms and life satisfaction (*p* < 0.05) (Table 2). At the second follow-up assessment, a positive relationship was found between changes in depressive symptoms and sleep quality perception (*p* < 0.05) (Table 2). Eight weeks after the intervention, results indicated a negative relationship between changes in anxiety symptoms and self-satisfaction with life (*p* < 0.05) (Table 2). At the final assessment, 12 weeks post-intervention, a negative relationship was also observed between life satisfaction and sleep quality (*p* < 0.05) (Table 2).

## 4. Discussion

The present pilot study investigated the prospective long-term advantages of integrating tDCS with individualized CBT in persons diagnosed with MDD. In accordance with our hypothesis and previous research, participants undergoing CBT plus active tDCS exhibited more significant reductions in depressive symptoms over time compared to those receiving CBT with sham stimulation. These effects became more pronounced during the follow-up period, particularly at 8 and 12 weeks, supporting the idea that the benefits of neuromodulation may build over time and persist after treatment ends.

Consistent with prior research indicating improved results from the combination of neuromodulation and pharmaceuticals in depression [17,18,19], our findings broaden this evidence to encompass psychological therapies, particularly CBT. Past research has consistently shown that combining active tDCS with antidepressants such as sertraline, escitalopram, or venlafaxine produces greater improvements than using either treatment alone. These findings suggest a potential synergistic effect of tDCS when combined with therapies targeting neurochemical or neurocognitive pathways. Nevertheless, the literature examining the synergistic potential of tDCS alongside psychotherapy is still very limited. Nejati et al. (2017) [20] conducted one of the few studies in this area, finding that the integration of tDCS with short-term psychodynamic psychotherapy resulted in significant and enduring decreases in depressive symptoms. Although promising, that study utilized a distinct therapeutic modality, thereby constraining the generalizability of its findings to evidence-based procedures such as CBT.

Alongside the primary goal of depression, the active tDCS group demonstrated incremental enhancements in sleep quality and anxiety symptoms, although these improvements did not achieve statistical significance at every assessment interval. Nonetheless, a trend of consistent improvement was noted, especially in subsequent evaluations. Furthermore, strong relationships between depressed symptoms and secondary domains—such as anxiety, sleep, and life satisfaction—indicate a cascading effect of symptom alleviation. These findings corroborate previous studies [21,22,23], suggesting that therapies aimed at core depressed symptoms may indirectly mitigate comorbid psychiatric issues. It is important to note that the correlation analyses rely on repeated measures, which may violate the assumption of independence. Although they provide exploratory insights into the temporal correlations among symptom dimensions, future research should utilize more rigorous longitudinal methodologies, such as multilevel modeling, to adequately address intra-subject variability. Consequently, our findings suggest that symptom alleviation in one domain (e.g., depression) may contribute to broader psychological improvements; however, this interpretation should be viewed as preliminary, given the exploratory nature of the correlation analyses.

These results are also provided in Figure 3, which shows that response and remission rates increased progressively over time for participants receiving active tDCS. By the last follow-up (T6), markedly more people in the active group clinically responded (defined as a minimum 12-point reduction in MADRS) and ended up in remission (BDI score ≤ 9). This affirms that the therapeutic benefit of the combined intervention is both sustained and accumulating. Also, correlational patterns showed in Table 2 showed consistent relationships with reduction in depressive symptoms and improvement of anxiety and sleep quality. This suggests that there might be some kind of dependency in symptom alleviation, where the early mitigation of depression benefits comorbid domains and, in turn, significantly expands the effects of treatment over time.

This study advances prior research by integrating CBT, acknowledged as a primary intervention for MDD due to its robust empirical backing and systematic, skills-oriented methodology. Our findings indicated that the integration of cognitive behavioral therapy (CBT) with active transcranial direct current stimulation (tDCS) led to a more significant reduction in symptoms, especially during the follow-up phase, implying that neuromodulation may augment the acquisition, retention, or application of cognitive and behavioral techniques acquired during therapy. This is particularly pertinent given the shared neurocognitive aims of both therapies, including the modulation of prefrontal cortex activity and enhancement of cognitive control over emotional reactions. The observation that these advantages were most significant at 8 and 12 weeks post-treatment further substantiates the concept that tDCS may promote neuroplastic alterations that reinforce and prolong the effects of CBT over time.

This pattern of postponed clinical benefit aligns with our previous research with tDCS in several domains. A randomized controlled experiment including persons with spinal cord injury shown that motor cortex (M1) stimulation led to a delayed reduction in pain symptoms, which became meaningful only after the conclusion of the stimulation phase [24]. The findings indicate that the therapeutic effects of tDCS may entail prolonged neurophysiological changes rather than instantaneous symptom alleviation, thereby elucidating the sustained improvements noted in the current trial. These combined observations support the notion that tDCS can enhance short-term therapeutic results and facilitate enduring alterations in brain function that persist after treatment concludes.

From a mechanistic standpoint, our findings substantiate the hypothesis that diminished activity in the left DLPFC, associated with deficiencies in emotion regulation, can be influenced by focused anodal stimulation [11,25,26]. The bilateral DLPFC montage employed in this investigation aligns with previous evidence affirming its effectiveness in improving cognitive control and emotional processing [27,28].The observation of improvements weeks after the cessation of stimulation indicates the potential for brain plasticity and the reinforcement of abilities learned through cognitive behavioral therapy via better prefrontal functioning.

### 4.1. Limitations

Despite the promising outcomes, this study presents numerous significant limitations that necessitate careful interpretation of the results. The limited sample size, resulting from recruiting and procedural difficulties during the COVID-19 pandemic, substantially restricted the statistical power of our analyses. Consequently, we could not utilize more advanced statistical techniques, such as linear mixed-effects models, which are more effective for identifying small temporal changes and managing missing data in longitudinal studies. This constraint may have resulted in type II errors, especially in the examination of secondary outcomes, when potentially significant trends did not achieve statistical significance. However, it is important to clarify that, as a pilot trial, the study aimed not to confirm efficacy but to assess feasibility, observe initial trends, and estimate effect sizes for planning future large-scale research.

In addition to the small sample size, the absence of a waitlist or treatment-as-usual control group limits our ability to isolate the specific effects of CBT from natural recovery or placebo influences. While steady medication use was allowed, the absence of categorization for pharmacological treatment presents possible confounding variables. The uniformity of our relatively youthful, CBT-naïve cohort with mild to moderate symptoms limits the generalizability to wider or more intricate clinical populations. Moreover, the 12-week follow-up may inadequately reflect the enduring benefits of the intervention. Ultimately, despite the collection of EEG data, the lack of neurobiological investigation in this publication precludes any inferences regarding the underlying processes of therapy success.

### 4.2. Implications for Future Research

Future studies should focus on incorporating bigger and more demographically varied samples, as well as multimodal outcome measures, to improve the interpretability, reliability, and generalizability of results. Integrating diverse participant groups will enable a more precise evaluation of treatment effectiveness across different clinical profiles and comorbidities.

Additionally, upcoming studies should explore alternative stimulation protocols—such as transcranial alternating current stimulation (tACS) and transcranial random noise stimulation (tRNS)—to optimize cortical engagement and therapeutic outcomes [29,30,31]. Emerging evidence also highlights the critical role of stimulation parameters (e.g., intensity, duration, frequency) in modulating brain activity, as demonstrated by EEG-indexed studies on transcranial pulsed current stimulation (tPCS) [32].

To personalize neuromodulatory interventions further, future work should prioritize the development and validation of neurophysiological and neuroimaging biomarkers. These biomarkers can support the real-time monitoring of brain responses and allow for the fine-tuning of stimulation protocols based on individual brain profiles. A recent comprehensive review by Irwin (2023) [33] emphasizes the need for such precision-based approaches in advancing the clinical utility of transcranial electrical stimulation.

## 5. Conclusions

This pilot study suggests that the integration of transcranial direct current stimulation (tDCS) and cognitive behavioral therapy (CBT) may improve treatment efficacy for patients with MDD, especially in the long-term relief of symptoms. While both groups were able to reap benefits from CBT, those who were given active tDCS were able to achieve even greater and longer-lasting reductions in depressive symptoms. Most notably, the benefits extended beyond depression, encompassing reduced anxiety, improved sleep, greater life satisfaction, and an enhanced sense of well-being, suggesting that the combined treatment may trigger a broader and progressively accelerating therapeutic effect.

These findings support the theory that neuromodulation of the dorsolateral prefrontal cortex enhances neuroplasticity and cognitive control, thereby amplifying the effects of CBT. The effects that were noted during follow-up assessments highlight the need to consider longer time frames, particularly in reference to measuring the effectiveness of multi-pronged therapy approaches.

Despite these drawbacks, the conclusions drawn from the study add strength to existing literature while simultaneously creating opportunities for further research. Future trials should include more diverse participant samples and incorporate neurobiological measures—such as EEG or neuroimaging—to better understand underlying mechanisms and refine treatment strategies. In turn, these results could help develop faster-acting, tailored treatment plans for individuals with major depressive disorder, especially those who do not respond well to traditional treatment methods.

## Figures and Tables

**Table 1:** Sociodemographic characteristics.

	Active Group (*n* = 6)	Sham Group (*n* = 4)	*U*/*χ*^2^	*df*	*p*
	*n* (%)	*n* (%)			
Age, *Mdn* [*IQR*]	31.00 [16.50]	31.50 [23.25]	10.00	-	0.670
Sex			0.28	1	0.598
Female	4 (66.7)	2 (50)			
Male	2 (33.3)	2 (50)			
Education			0.63	1	0.429
High school	3 (50)	3 (75)			
Higher education	3 (50)	1 (25)			
Marital status			2.86	2	0.240
Single	3 (50)	4 (100)			
Married/non-marital partnership	1 (16.7)	-			
Divorced	2 (33.3)	-			
Work status			1.88	2	0.392
Student	-	1 (25)			
Employed	3 (50)	2 (50)			
Unemployed	3 (50)	1 (25)			

**Table 2:** Correlations between depression, anxiety, sleep, and life satisfaction over time. Significant correlations are marked: *p* < 0.05 (*), *p* < 0.01 (**).

	BDI	BAI	STAI-Y	PSQI	SWLS
**T0**					
MADRS	0.09	0.14	−0.16	0.38	−0.13
BDI	-	0.30	0.30	0.47	−0.46
BAI	-	-	0.03	0.38	−0.43
STAI-Y	-	-	-	−0.04	−0.06
PSQI	-	-	-	-	−0.28
**T1**					
MADRS	0.59	0.37	0.03	0.15	0.20
BDI	-	−0.18	0.03	0.18	0.35
BAI	-	-	−0.45	−0.02	0.26
STAI-Y	-	-	-	−0.31	−0.56
PSQI	-	-	-	-	−0.20
**T2**					
MADRS	0.80 *	0.33	0.07	0.49	−0.09
BDI	-	0.42	0.69 *	0.43	−0.52
BAI	-	-	0.14	0.59	−0.26
STAI-Y	-	-	-	0.20	−0.75 *
PSQI	-	-	-	-	−0.34
**T3**					
MADRS	0.87 **	0.55	0.08	0.25	0.55
BDI	-	0.61	0.44	−0.33	0.65 *
BAI	-	-	0.64 *	−0.27	0.58
STAI-Y	-	-	-	−0.63	0.22
PSQI	-	-	-	-	−0.05
**T4**					
MADRS	0.55	0.44	0.75 *	0.02	−0.35
BDI	-	0.55	0.45	0.64 *	−0.06
BAI	-	-	0.68 *	0.55	−0.08
STAI-Y	-	-	-	0.05	−0.43
PSQI	-	-	-	-	−0.08
**T5**					
MADRS	0.56	0.59	0.73 *	−0.19	−0.44
BDI	-	0.66 *	0.34	−0.37	−0.43
BAI	-	-	0.53	−0.20	−0.27
STAI-Y	-	-	-	0.18	−0.76 *
PSQI	-	-	-	-	−0.38
**T6**					
MADRS	0.78 *	0.51	0.94 *	0.25	−0.34
BDI	-	0.67 *	0.68 *	0.14	−0.31
BAI	-	-	0.57	0.29	−0.48
STAI-Y	-	-	-	0.54	−0.60
PSQI	-	-	-	-	−0.63 *

## Data Availability

The data supporting the findings of this study are available from the corresponding author upon reasonable request. Due to ethical and privacy restrictions related to sensitive clinical information, the dataset is not publicly available.

## Abbreviations

The following abbreviations are used in this manuscript:

**Abbreviation**	**Definition**
MDD	major depressive disorder
CBT	cognitive behavioral therapy
tDCS	transcranial direct current stimulation
DLPFC	dorsolateral prefrontal cortex
MADRS	Montgomery–Åsberg Depression Rating Scale
BDI	Beck Depression Inventory
BAI	Beck Anxiety Inventory
STAI	State-Trait Anxiety Inventory
PSQI	Pittsburgh Sleep Quality Index
SWLS	Satisfaction With Life Scale
EEG	electroencephalogram
SCID-5	Structured Clinical Interview for DSM-5
SECVS	Subcommittee on Ethics for Life and Health Sciences
RCT	randomized controlled trial
